# Functional determinism amid taxonomic stochasticity: insights into rules governing the assembly of algal-microbial symbioses

**DOI:** 10.1128/aem.00359-26

**Published:** 2026-03-31

**Authors:** Tian Deng, Huan Wang, Shu-Feng Zhang, Xin-Yao Wu, Ze-Sheng Yang, Da-Zhi Wang, Yue Zheng

**Affiliations:** 1 State Key Laboratory of Marine Environmental Science, College of the Environment and Ecology, Xiamen University 12466, Xiamen, China; 2 Fujian Ocean Innovation Center, Xiamen, China; 3 Key Laboratory of the Ministry of Education for Coastal and Wetland Ecosystems, Xiamen University 12466, Xiamen, China; Georgia Institute of Technology, Atlanta, Georgia, USA

**Keywords:** phycosphere microbiota, algal-microbial symbiosis, deterministic and stochastic assembly, functional redundancy, *Skeletonema*

## Abstract

Marine algal-microbial symbioses constitute essential functional units that drive ocean biogeochemical cycles and trigger harmful algal blooms. Yet, a long-standing controversy persists regarding the mechanisms of algal-microbial symbiose assembly, specifically whether phycosphere microbiota are predominantly shaped by deterministic algal-driven selection or by stochastic environmental processes, with no definitive resolution to date. Here, we examined phycosphere communities associated with a series of *Skeletonema* strains, tracking their taxonomic and functional dynamics across successive growth stages. Despite pronounced taxonomic diversity, reflected in distinct community compositions, successional trajectories, and microbial networks, shotgun metagenomic analyses revealed highly conserved functional repertoires across samples, with consistently abundant core pathways, including amino acid biosynthesis, secondary metabolite and antibiotic production, and ABC transport systems. Statistical analyses further revealed a marked decoupling of taxonomy and function, with functional redundancy enabling taxonomically distinct lineages to perform equivalent metabolic roles. Based on these findings, we propose a dual assembly model in which deterministic algal host-driven selection constrains functional composition, while stochastic processes govern species-level membership. This “function-first, taxonomy-stochastic” paradigm reconciles opposing assembly theories, underscores functional resilience in the face of taxonomic turnover, and provides a conceptual foundation for the rational design of synthetic algal-microbial consortia in marine biotechnological applications.

**IMPORTANCE:**

Marine algae live in close association with diverse microorganisms that influence nutrient cycling and ecosystem stability. Yet, how these algal-microbial partnerships assemble and maintain functional integrity remains unresolved. Using *Skeletonema* as a model, this study demonstrates that while the microbial species surrounding different algal strains vary greatly, their metabolic functions remain remarkably consistent. This finding reveals that algal hosts deterministically shape the functional needs of their microbiome, whereas the specific bacterial members fulfilling those roles are interchangeable. Such a “function-first” organization explains how algal-microbial symbioses persist despite environmental fluctuations. Understanding these assembly rules not only advances our knowledge of marine microbial ecology but also provides a conceptual foundation for engineering stable and resilient algal-microbial consortia for sustainable ocean biotechnologies.

## INTRODUCTION

Marine algal-microbial symbioses constitute functional units comprising algae and closely associated microbial communities, playing indispensable roles in marine biogeochemical cycles and harmful algal blooms (1–3). These algal-microbial consortia establish efficient internal nutrient-recycling systems, enabling symbiotic entities to sustain high cell densities and vigorous metabolic activities even in oligotrophic marine regions (4, 5). The ecological significance of these symbiotic interactions is primarily manifested in the cycling of carbon (6), nitrogen (5, 7), phosphorus (8), and other elements (3, 9), significantly influencing global climate regulation (10). Additionally, algal-microbial symbioses markedly regulate key ecological phenomena, including harmful algal blooms (11–13) and coral bleaching events (14–16). As a critical bridge linking individual organisms to ecosystem-level communities, in-depth exploration of marine algal-microbial symbioses is pivotal to integrating marine physiology with marine ecology.

Research on algal-microbial interactions has transitioned from describing consortium structure and function to elucidating interaction mechanisms, propelled by innovations in omics technologies and *in situ* methodologies (17–19). Currently, beyond clarifying interaction mechanisms, a fundamental scientific question emerges: What rules govern the assembly of algal-microbial symbioses? Regarding mechanisms underlying the assembly of algal-microbial consortia, two competing theoretical frameworks dominate discussions, centering on the importance of deterministic processes (algal selection) versus stochastic processes (environmental randomness). The deterministic-dominated perspective emphasizes that algal metabolic traits and chemical signaling exert strong selective pressures on phycosphere microbiota, recruiting relatively conserved core microbial groups (20, 21). For instance, clear algal-specific community structures were found at both the family and genus levels, through comparing microbial communities associated with *Phaeodactylum tricornutum* and *Microchloropsis salina* across environment mesocosms and laboratory enrichments (22). Similarly, the *Synechococcus* and phycosphere microbial communities form mutualistic networks of nitrogen fixation and carbon exchange, with phycosphere community succession driven by the stoichiometric characteristics of dissolved organic carbon released by *Synechococcus* algae (4). Such evidence indicates targeted phycosphere microbial recruitment via algal-mediated “chemical signaling.”

Conversely, the stochastic-dominated viewpoint posits that abiotic factors, including physicochemical environmental conditions, dispersal limitations, and random colonization, predominantly shape phycosphere microbiota composition. For instance, modeling studies of free-living *Symbiodiniaceae* communities indicate that stochastic processes, including dispersal limitation and ecological drift, primarily govern community assembly, with weaker explanatory power attributed to host selection (23). Similarly, recent experimental evidence from Southern Ocean phytoplankton microbiomes demonstrates that although phytoplankton exert selective pressure on microbial community assembly, stochastic drift remains the dominant force shaping community structure across growth phases (24). Exogenous nutrient additions can randomly perturb established phycosphere microbiota, disrupting pre-existing mutualistic networks (25). Although symbiotic relationships may be re-established post-nutrient depletion, reassembly trajectories are influenced by stochastic microbial behaviors (e.g., chemotaxis, quorum sensing), often resulting in community compositions that diverge from original states. This non-deterministic reassembly implies environmental disturbances can reset community assembly pathways.

The current debate revolves around whether the assembly of algal-microbial symbioses is predominantly driven by deterministic algal-intrinsic factors or governed by stochastic environmental processes. To address this critical question, the phycosphere microbiota of multiple *Skeletonema* strains freshly isolated from coastal waters were systematically examined. Physiological traits were first assessed, confirming comparable growth and nutrient uptake capacities across strains. Subsequently, the phycosphere microbial communities were characterized with respect to taxonomic composition and the distinction between core and strain-specific members. Community succession across growth stages was further analyzed, and co-occurrence networks were constructed to elucidate interspecies interaction patterns. Finally, functional potentials were evaluated to determine whether metabolic capabilities are conserved despite taxonomic diversity. Collectively, these multi-dimensional analyses establish a comprehensive framework for disentangling deterministic functional selection from stochastic taxonomic processes in algal-microbial symbioses.

## RESULTS

### Different *Skeletonema* strains show similar physiological phenotypes

**Fig 1:**
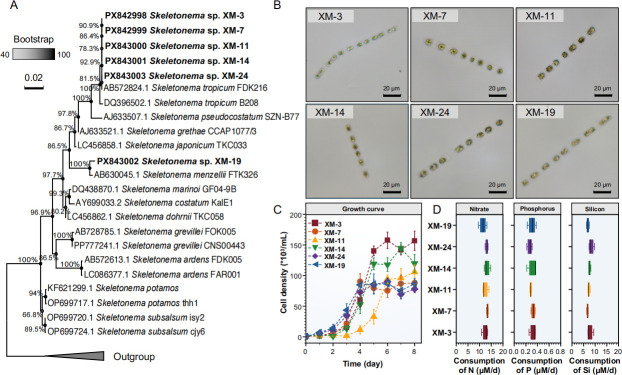
Phylogenetic and physiological characteristics of different *Skeletonema* strains. (**A**) Neighbor-joining phylogenetic tree of *Skeletonema* based on LSU rDNA sequences, with bootstrap support values calculated from 1,000 replicates. *Thalassiosira* spp., *Planktoniella tubulata*, and *Stephanodiscus suzukii* were included as outgroups within the same order. (**B**) Morphology of six *Skeletonema* strains observed under an optical microscope. (**C**) Growth curves of six *Skeletonema* strains. (**D**) Inorganic nutrient consumption rate of the six *Skeletonema* strains.

*Skeletonema* is a globally distributed diatom genus in marine environments (26). *Skeletonema* algae and its surrounding microorganisms serve as a vital food source for mariculture organisms (e.g., shellfish) and are also primary contributors to marine ecological events, including red tides. As a typical marine diatom, to resolve the structure of algae-microbial symbionts, six *Skeletonema* strains (designated XM-3, XM-7, XM-11, XM-14, XM-19, and XM-24) were freshly isolated from the coastal waters of Xiamen, China, for this study. Phylogenetic analysis based on the LSU rDNA gene (Fig. 1A) revealed that strains XM-3, XM-7, XM-11, XM-14, and XM-24 clustered closely with two reference *Skeletonema tropicum* strains (FDK216 and B208), with sequence identities greater than 99%. This high sequence similarity supports their identification as *S. tropicum*. In contrast, strain XM-19 clustered with the *Skeletonema menzelii* reference strain FTK326, with a sequence identity of 98.98%, indicating its classification as *S. menzelii*.

All isolated *Skeletonema* strains formed chains composed of 2–20 cells. Individual cells measured approximately 4–6 μm in width and 5–8 μm in length (Fig. 1B; Table S1). Furthermore, the six strains exhibited comparable physiological traits, including growth rates and patterns of nitrogen (N), phosphorus (P), and silicon (Si) consumption (Fig. 1C and D). The doubling times ranged from about 0.53 to 0.69 days. Nutrient consumption rates were measured as follows: nitrogen (11.4–13.4 μmol/L/day), phosphorus (0.27–0.33 μmol/L/day), and silicon (6.7–8.3 μmol/L/day). Statistical analysis indicated no significant differences in these metabolic rates among the strains. The consistency in physiological traits among the six strains indicates comparable metabolic capabilities and ecological strategies for resource acquisition and growth regulation.

### Phycosphere microbial composition of different *Skeletonema* strains

**Fig 2:**
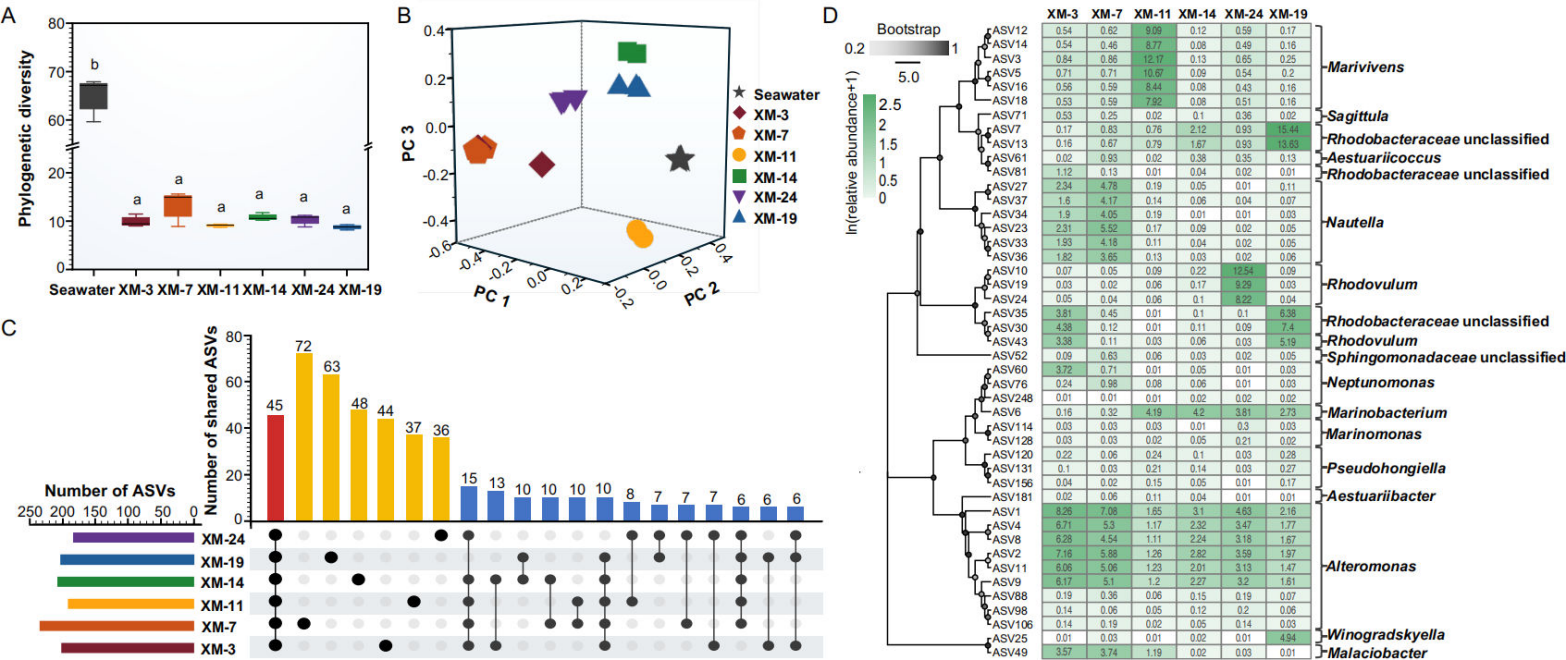
Phycosphere microbial community composition associated with six *Skeletonema* strains. (**A**) Alpha diversity of phycosphere microbial communities in the six *Skeletonema* strains and the ambient seawater. (**B**) Beta diversity of phycosphere microbial communities in the six *Skeletonema* strains and ambient seawater (based on Bray-Curtis PCoA). (**C**) UpSet plot showing shared and unique ASVs across the six *Skeletonema* phycospheres. Vertical bars represent the number of shared ASVs among strains (denoted by connected circles below), red bars indicate ASVs shared by all strains, yellow bars indicate strain-specific ASVs, and horizontal bars represent the total ASV set size for each strain. (**D**) Phylogenetic relationship of shared ASVs based on 16S rRNA gene sequence similarity, along with their average relative abundances across the *Skeletonema* strains (heatmap values represent average relative abundance).

To characterize the phycosphere microbiota of *Skeletonema* strains, amplicon sequencing revealed 562 amplicon sequence variants (ASVs) spanning 114 genera and 10 classes across all *Skeletonema* strains. This represents a substantial reduction compared to the approximately 6,000 ASVs detected in ambient seawater *in situ*, indicating selective microbial enrichment within the phycosphere microenvironment distinct from the surrounding seawater environment. Alpha diversity analysis revealed that microbial diversity within the *Skeletonema* phycosphere was significantly lower than that of the ambient seawater (Fig. 2A; Fig. S1A and B). Similarly, PCoA based on Bray-Curtis and Jaccard dissimilarities showed clear separation in microbial community composition between the *Skeletonema* phycosphere and the ambient seawater (Fig. 2B; Fig. S1C). This further underscores the deterministic influence of diatom strain identity on phycosphere community structure.

Core and strain-specific features of the *Skeletonema* phycosphere microbiota were subsequently characterized. Alpha diversity analysis revealed no significant differences in richness or phylogenetic diversity among the six phycosphere communities (Fig. 2A; Fig. S1A and B). Analysis of shared microbial taxa identified 45 core ASVs present across all six strains (Fig. 2C), comprising 18.5%–23.6% of each community (Fig. S3). At the ASV level, this conserved core was primarily represented by ASVs affiliated with genera including *Marivivens*, *Nautella*, *Rhodovulum*, *Alteromonas*, and unclassified genera of *Rhodobacteraceae* (Fig. 2D). However, this divergence was corroborated by PCoA, which showed clear separation among all *Skeletonema* strains except for XM-3 and XM-7 (Fig. 2B and Fig. S1C). Moreover, taxonomic composition exhibited dissimilarities among different *Skeletonema* strains, especially at the genus level (Fig. S2). At the ASV level, 19%–31% of ASVs in each strain were strain-specific, but their cumulative relative abundances were low, accounting for less than 6% of the community (Fig. S3). The remainder of the ASVs were accessory taxa shared by subsets of strains (>1 but <6), yet they contributed substantially to community structure. Notably, even shared ASVs exhibited significant inter-strain abundance variations (Fig. 2D; Fig. S4). LEfSe analysis (LDA > 3.0, *P* < 0.01; Fig. S5) identified multiple differentially enriched taxa across the six phycospheres, including ASVs affiliated with several bacterial genera (e.g., *Sagittula*, *Flavobacterium*, *Alteromonas*), among others. These findings indicate substantial heterogeneity in phycosphere microbiota among closely related algal strains, seemingly supporting stochastic processes in phycosphere community assembly despite high host similarity.

### Dynamics of *Skeletonema* phycosphere microbiota across growth stages

**Fig 3:**
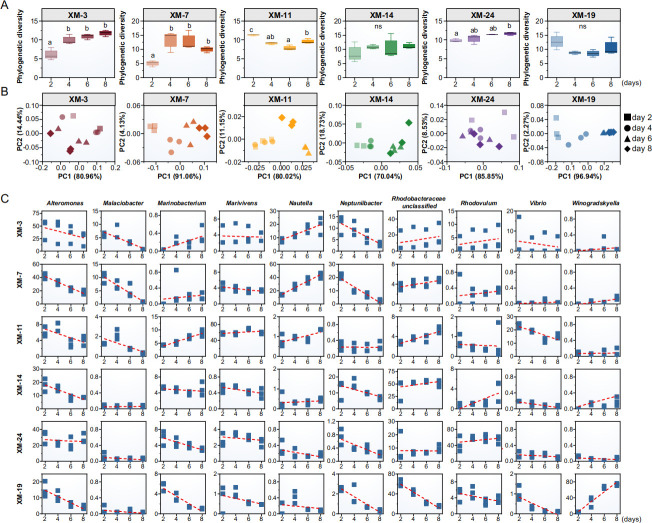
Dynamics of phycosphere microbial communities across growth stages of six *Skeletonema* strains. (**A**) Alpha diversity of phycosphere microbial communities across different growth stages. (**B**) Beta diversity of phycosphere microbial communities across different growth stages (based on Bray-Curtis PCoA). (**C**) Relative abundance of major microbial genera (top 10) at different growth stages, with red dotted lines representing fitted curves showing temporal trends in community shifts

The phycosphere microbiota exhibited dynamic temporal changes rather than maintaining a static composition. To elucidate these successional patterns, we characterized the microbiota associated with four growth stages (sampling was conducted at days 2, 4, 6, and 8 to represent consecutive cultivation time points, rather than discrete classical growth phases) of *Skeletonema* strains. Alpha diversity exhibited strain-dependent trajectories, such as the phylogenetic diversity increasing progressively in XM-3, XM-7, and XM-24 cultures, decreasing in XM-11, and remaining relatively stable with minimal fluctuations in XM-14 and XM-19 (Fig. 3A). Similarly, richness and evenness also exhibited distinct successional patterns across different *Skeletonema* strains (Fig. S6). PCoA results revealed significant temporal shifts in community structure for strains XM-3, XM-7, XM-11, XM-14, and XM-19, while the community composition of XM-24 remained relatively stable over time (Fig. 3B; Table S2).

Taxonomic analysis further confirmed divergent successional dynamics at the genus level (Fig. 3C). Only a few taxa (e.g., *Malaciobacter*, *Neptuniibacter, Winogradskyella*) exhibited conserved temporal dynamics across all strains, showing a consistent increase or decrease with the growth of *Skeletonema*. In contrast, most genera displayed strain-specific successional patterns. For instance, *Alteromonas* remained relatively stable during XM-24 but declined in the other five strains. Unclassified genera within *Rhodobacteraceae* increased in XM-3, XM-7, XM-11, and XM-14, but decreased in XM-19. Other dominated genera, such as *Marinobacterium*, *Marivivens*, *Nautella,* and *Rhodovulum*, also showed strain-specific successional trends. These results indicate that phycosphere microbial communities differ not only in taxonomic composition but also in their temporal assembly patterns across *Skeletonema* strains.

### Co-occurrence network analysis of *Skeletonema* phycosphere microbiota

**Fig 4:**
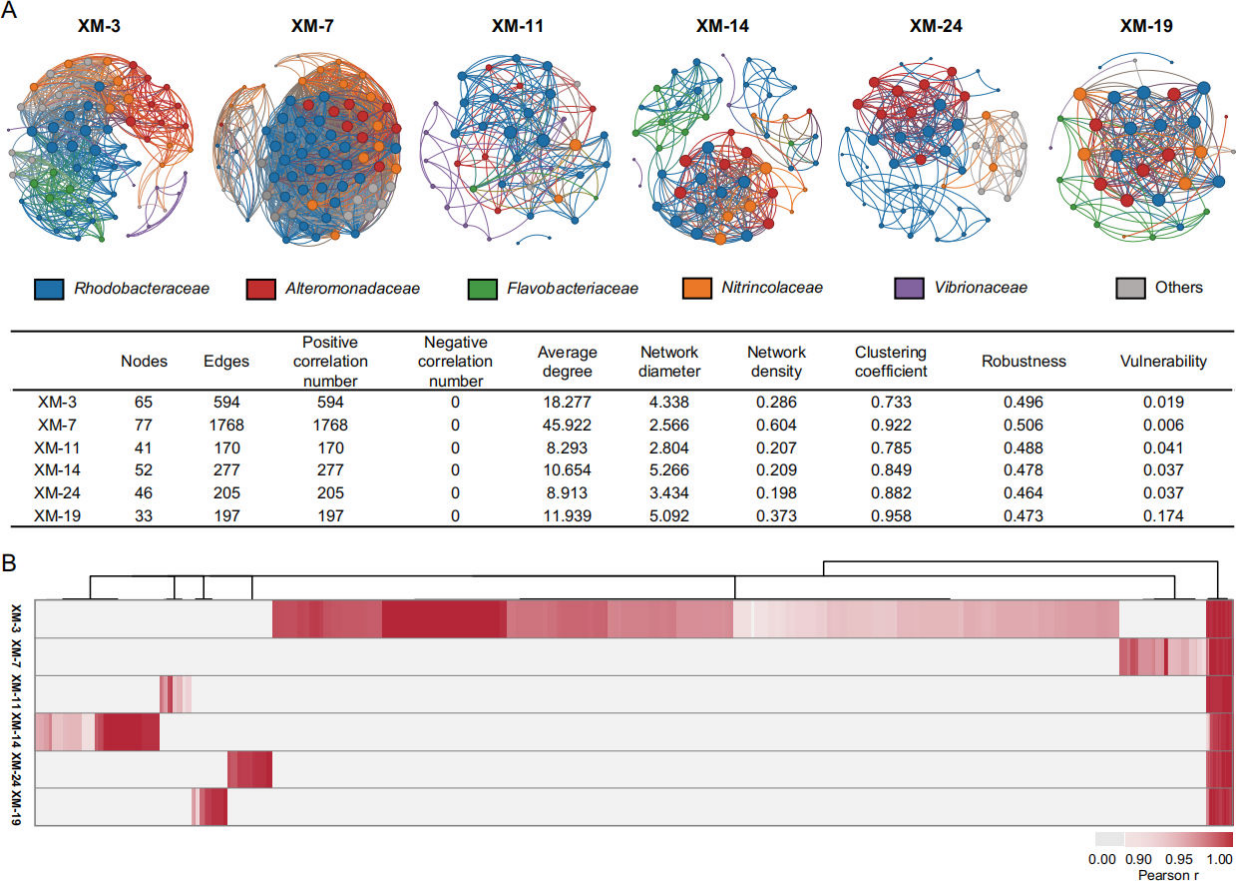
Co-occurrence networks of phycosphere microbiota associated with six *Skeletonema* strains. (**A**) Co-occurrence networks constructed at the ASV level. Each node represents a unique ASV, with node color indicating its family-level taxonomic affiliation, and node size proportional to degree. (**B**) Heatmap of ASVs interaction weights within the co-occurrence network (*r* > 0.9, *P* < 0.01).

Co-occurrence network analysis revealed pronounced structural differences in phycosphere microbial communities among the six *Skeletonema* strains (Fig. 4A). XM-7 formed the largest and most highly integrated network (77 nodes, 1,768 edges) characterized by the highest average degree (45.92), density (0.604), and clustering coefficient (0.922). In contrast, XM-19 exhibited the smallest network (33 nodes) but the highest density (0.373) and clustering coefficient (0.958), reflecting a compact, hub-centric architecture. All networks showed robustness values ranging from 0.46 to 0.51 after random removal of 50% of nodes, indicating a relatively high tolerance to random node removal in the context of ecological network analyses. However, vulnerability differed greatly, being lowest in XM-7 (0.006) and highest in XM-19 (0.174), suggesting that XM-19 was highly dependent on a few hub nodes and therefore prone to collapse under targeted attacks despite its high robustness to random perturbations. Additionally, all networks were exclusively composed of positive correlations, suggesting mutualistic or cooperative interactions dominated the phycosphere microbiomes.

Further analysis of high-weight interactions (Spearman’s rho correlation coefficient > 0.9) at ASV level revealed that, although some potential interactions were shared across strains, the majority were strain-specific (Fig. 4B). Among them, XM-3 displayed the greatest number of unique interactions, primarily involving families such as *Alteromonadaceae*, *Rhodobacteraceae*, *Burkholderiaceae*, *Nitrincolaceae*, and *Sphingomonadaceae*. In contrast, XM-11 showed significantly fewer potential interactions, which were largely dominated by associations between *Rhodobacteraceae* and *Vibrionaceae*. Distinct interaction patterns were also observed in XM-7, XM-14, XM-24, and XM-19. These results further highlight the structural and functional specificity of microbial interaction networks within the phycosphere of different *Skeletonema* strains.

### Functional conservation amid taxonomic diversity in *Skeletonema* phycosphere microbiota

**Fig 5:**
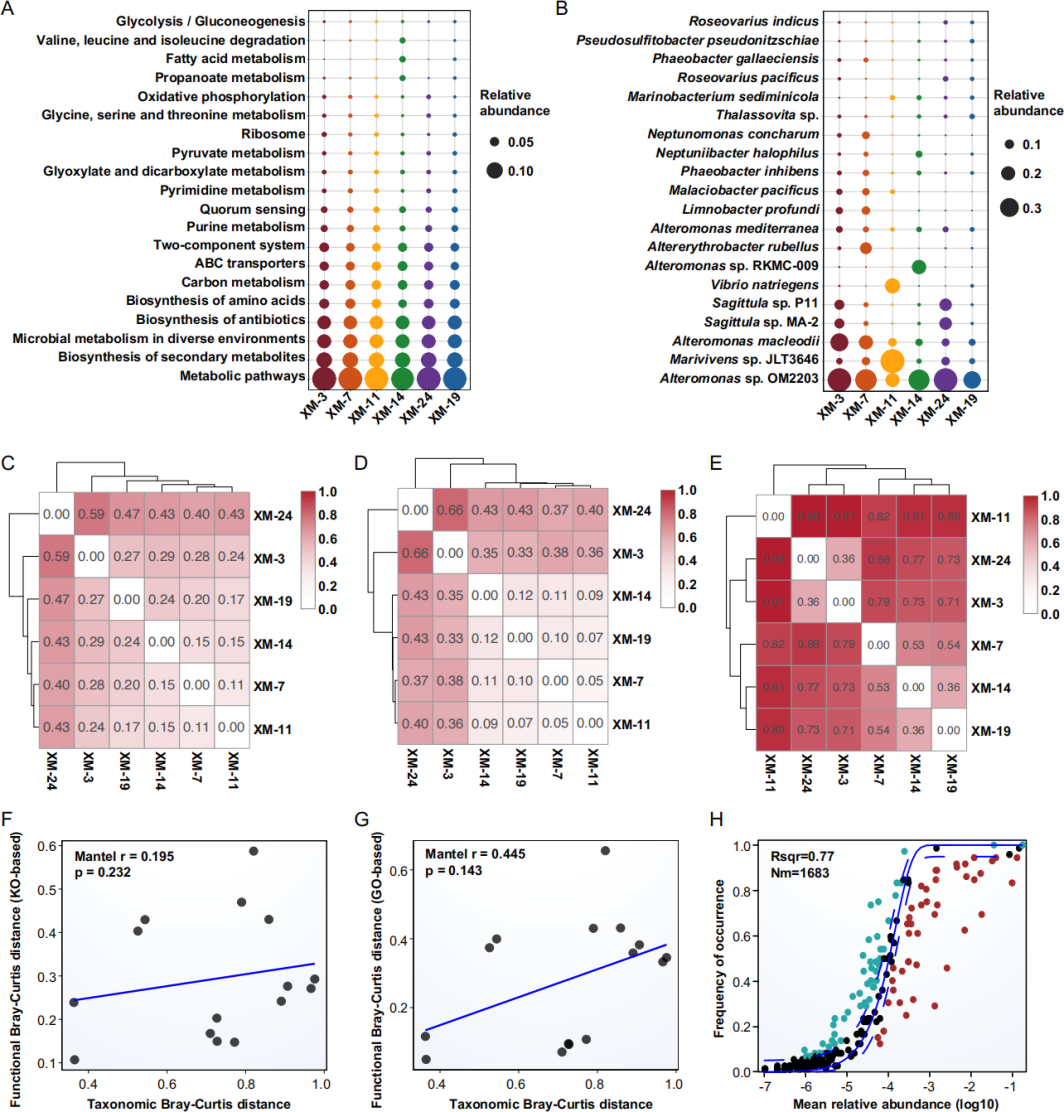
Functional and taxonomic profiles of phycosphere microbiota in six *Skeletonema* strains. (**A**) Relative abundance of the top 20 KEGG pathways. The “top 20” pathways were selected based on the total relative abundance summed across all samples. (**B**) Relative abundances of the top 20 microbial genera, ranked by their total relative abundance across all samples. (**C**) Functional differences (KO-based) among different samples based on Bray-Curtis distance. (**D**) Functional differences (GO-based) among different samples based on Bray-Curtis distance. (**E**) Taxonomic differences among different samples based on Bray-Curtis distance. (**F**) Mantel test correlation between functional Bray-Curtis distance (KO-based) and taxonomic Bray-Curtis distance. (**G**) Mantel test correlation between functional Bray-Curtis distance (GO-based) and taxonomic Bray-Curtis distance. (**H**) Neutral community model fitting of phycosphere community assembly.

Metagenomic analysis revealed a high degree of functional conservation within the phycosphere microbiota across different *Skeletonema* samples. A core set of metabolic pathways was consistently abundant, including the biosynthesis of secondary metabolites, antibiotics, and amino acids, as well as a high abundance of ABC transporters (Fig. 5A). In contrast, the taxonomic composition of these communities exhibited considerable heterogeneity (Fig. 5B). This functional-taxonomic dichotomy was further supported by Bray-Curtis dissimilarity analysis, which demonstrated significantly lower inter-sample variability in both KEGG Orthology (KO)- and Gene Ontology (GO)-based functional profiles (Fig. 5C and D) compared to the pronounced dissimilarities observed in taxonomic composition (Fig. 5E). Collectively, these findings confirm that while the taxonomic makeup of the phycosphere microbiota varies substantially, its core functional repertoire remains remarkably conserved across different *Skeletonema* hosts. Furthermore, Mantel tests revealed no significant correlation between taxonomic composition and functional profiles, whether based on KO (*P* = 0.232) or GO annotations (*P* = 0.143) (Fig. 5F and G). This functional-taxonomic decoupling suggests that the ecological role of the phycosphere microbiome is maintained by different bacterial taxa performing similar metabolic functions, supporting the principle of functional redundancy within this symbiotic system.

Similarly, the functional profiles predicted based on the 16S rRNA sequence yielded consistent results: the functions of the phycosphere microbiota were highly conserved among different *Skeletonema*, while taxonomic composition exhibited pronounced heterogeneity (Fig. S7 and S8). Inter-strain divergence intensified with increasing taxonomic resolution, becoming most evident at the genus level (Fig. S8 and S9). The genus level displayed significant variation both across strains and throughout growth stages, indicating strain-dependent assembly at fine taxonomic scales. ASV-level taxonomic PCoA based on Bray-Curtis distances demonstrated clear inter-strain separation (Fig. S10A). In contrast, KO-based functional PCoA showed convergence among most samples, with divergence only in late-stage XM-19 cultures (Fig. S10B). Statistical comparisons confirmed greater taxonomic dissimilarity: ANOSIM (*r* = 0.951, *P* = 0.001) and PERMANOVA (*R*^2^ = 0.801, F = 53.17, *P* = 0.0001) values for taxonomic composition substantially exceeded those for functional profiles (ANOSIM R = 0.667, *P* = 0.001; PERMANOVA *R*^2^ = 0.617, *F* = 21.29, *P* = 0.0001). Jaccard-based analyses further corroborated this pattern (Fig. S10C and D): taxonomic composition differed significantly (ANOSIM *R* = 0.648, *P* = 0.001; PERMANOVA *R*^2^ = 0.247, *F* = 4.32, *P* = 0.0001), while KO-based functional composition showed no significant differences (ANOSIM R = 0.0939, *P* = 0.001; PERMANOVA *R*^2^ = 0.102, *F* = 1.51, *P* = 0.087).

Neutral community model analysis indicated that the phycosphere microbial community was largely shaped by stochastic processes (*R*^2^ = 0.77, Fig. 5H). The fitted dispersal parameter (*Nm* = 1,683) suggests a high effective dispersal rate in the sense of the neutral model, that is, a high probability that local taxa are replaced by individuals drawn from the regional metacommunity. This indicated that, despite significant variability in the phycosphere microbial community composition among different *Skeletonema* strains, the high effective dispersal rate contributed to maintaining the stability of its functional performance.

Collectively, these findings establish that while *Skeletonema* phycosphere microbiota exhibit taxonomic diversity, their functional profiles remain conserved. We propose that algal-microbial symbiont assembly is governed by metabolic functional requirements. Given functional redundancy across microbial taxa, stochastic processes may drive species-level variation once core metabolic functions are fulfilled.

## DISCUSSION

### Rules for assembly of algae-microbial symbionts

This study provides novel empirical evidence that the assembly of phycosphere microbiota is characterized by taxonomic diversity but functional convergence. Across different phycospheres of *Skeletonema* hosts, microbial species exhibited substantial variation in composition and successional dynamics, whereas the functional profiles were highly conserved. This functional convergence indicates that the metabolic requirements of the algal host exert strong deterministic selection on the functional traits of associated microbiota, while the specific microbial taxa fulfilling these functions are interchangeable. Consequently, the species-level assembly appears stochastic. This finding directly addresses the long-standing debate between deterministic and stochastic assembly theories in algal-microbial symbioses, revealing a dual-pattern mechanism in which functional assembly is deterministic but taxonomic assembly is substantially stochastic. In the following discussion, we compare these findings with patterns observed in other cross-kingdom symbiotic systems, explore the underlying ecological logic, and highlight the theoretical and applied implications.

### Ecological mechanisms: Functional redundancy and host selection

Theoretical interpretation of these findings is critical for understanding how cross-kingdom symbiotic systems are structured in marine environments. The phycosphere represents a microscale yet highly specialized niche in which the metabolic exchanges between algal hosts and microorganisms are intense, selective, and spatially confined (1). Such a microenvironment imposes strong constraints on the range of microbial functions that can be stably maintained, leading to a relatively low functional diversity threshold compared with open-ocean microbial assemblages. Once this functional “requirement ceiling” is met, multiple taxa with overlapping metabolic capabilities can coexist or replace each other, leading to high taxonomic variability. This scenario is consistent with the functional redundancy framework (27), whereby the loss of one taxon can be compensated by other taxa performing equivalent roles, thereby buffering functional stability against taxonomic turnover. In our study, this redundancy is exemplified by the dominance of members of the *Alteromonadaceae*, *Rhodobacteraceae*, and *Flavobacteraceae* within *Skeletonema* phycospheres. These microbial groups, frequently reported as major constituents of phytoplankton-associated microbiota and known to form close symbiotic relationships with algal hosts, exhibit considerable metabolic diversity, broad substrate utilization spectra, and strong environmental adaptability (28–30). Importantly, their high functional similarity means that despite substantial variations in community composition among algal hosts, the overall functional profile of the phycosphere remains conserved. This provides a mechanistic explanation for how deterministic functional filtering by the algal host can be maintained alongside high taxonomic turnover.

The conceptual framework we propose (Fig. S11) links host population size and complexity to patterns of functional and taxonomic diversity in associated microbiota. As the number of hosts of the same species increases, functional diversity of the associated microbiome approaches saturation more rapidly than taxonomic diversity. Beyond this saturation point, additional hosts can still harbor new taxa, but these taxa are functionally redundant. In the case of *Skeletonema*, the constrained complexity of the phycosphere environment means that the set of required microbial functions is relatively small and easier to satisfy than in the more heterogeneous open-ocean environment. Consequently, functional composition is highly conserved across hosts, whereas taxonomic composition is more susceptible to stochastic processes such as ecological drift, priority effects, and probabilistic dispersal. This framework reconciles deterministic and stochastic perspectives: deterministic selection operates at the level of functions, while stochasticity dominates at the level of taxa.

Our results are consistent with growing evidence that algal-associated microbiomes often exhibit high taxonomic variability but stable functional profiles, a pattern indicative of strong functional redundancy. For example, in the green macroalga *Ulva australis*, highly variable microbial communities maintain similar functional profiles (31, 32); this pattern is further supported by a recent large-scale study showing that Ulva microbiomes undergo substantial taxonomic turnover across a 2,000 km salinity gradient while exhibiting minimal functional shifts, demonstrating that different bacterial taxa can fulfill comparable ecological roles within the phycosphere (33). In the brown alga *Ectocarpus*, although bacterial community composition varies significantly across salinity regimes, the predicted metagenomic functions remain consistent, suggesting functional redundancy within the phycosphere microbiome (34). Additionally, comparable patterns have been documented in other systems: in plant rhizosphere and phycosphere microbiota, host-specific metabolic requirements also lead to functional convergence despite taxonomic diversity (35, 36); and in soil, environmental filters on function allow wide taxonomic variability to persist (37, 38). Taken together, these findings support the notion that algal-associated microbiomes may be assembled following a “function-first, taxonomy-stochastic” principle, in which functional traits are strongly selected by the host, while taxonomic composition is more influenced by stochastic processes. Our results suggest that this principle may have a certain degree of generality. However, given the high complexity and diversity of marine algae, the assembly mechanisms of phycosphere microbiomes could vary substantially among different algal taxa. Future comparative studies across a broader phylogenetic range of algal hosts and different environmental contexts are needed to elucidate the general principles governing the assembly of algal-microbial consortia.”

### Ecological and applied implications

From a broader perspective, understanding the assembly rules of algal-microbial symbioses has both fundamental and applied significance. In natural marine ecosystems, algal-microbial consortia regulate elemental cycles of carbon, nitrogen, and other key nutrients, thereby contributing to global biogeochemical regulation and affecting processes including oxygen production, CO_2_ drawdown, and the onset of ecological events such as harmful algal blooms and coral bleaching (3, 11, 12, 14). From an applied standpoint, insights into functional redundancy and deterministic host-driven functional filtering can inform the rational design of synthetic algal-microbial consortia for biotechnological purposes, including carbon sequestration, water quality management, and biofuel production (39). If functional requirements can be met by a variety of taxa, engineered consortia could be optimized for resilience and efficiency by selecting microbial partners that are both functionally compatible and tolerant to environmental fluctuations.

Nevertheless, our study has limitations that point to future research directions. First, while our findings are robust for the *Skeletonema* strains examined, the generality of the deterministic-functional/stochastic-taxonomic assembly model requires validation across a wider range of phytoplankton hosts and environmental conditions. Second, metagenomic, metatranscriptomic, and metabolomic analyses could provide a more direct and comprehensive view of functional expression and metabolite exchange within the phycosphere. Third, while we interpret taxonomic diversity as primarily driven by stochastic processes under conditions of high functional redundancy, targeted experiments manipulating colonization sequences, dispersal rates, and environmental variability could enable explicit quantification of stochastic versus deterministic contributions to community assembly. In summary, this study elucidates a dual assembly pattern in the *Skeletonema* phycosphere microbiota, where deterministic processes govern functional convergence and stochastic processes drive taxonomic diversity. This “function-first, taxonomy-stochastic” framework not only reconciles opposing views on microbial community assembly but also provides a conceptual basis for predicting microbiome responses to environmental change. Future work could harness this knowledge to design minimal, functionally efficient synthetic algal-microbial consortia tailored for ecological restoration and industrial applications, thereby translating ecological theory into practical solutions.

### Conclusion

This study reveals that *Skeletonema* phycosphere microbiota exhibit pronounced taxonomic diversity yet striking functional convergence, reflecting a dual assembly mechanism. Deterministic host selection constrains the functional traits of associated microorganisms, ensuring that essential metabolic pathways are consistently maintained. Within this functional framework, high redundancy allows diverse microbial taxa to occupy equivalent ecological niches, leading to stochastic variation in species composition. This pattern reconciles deterministic and stochastic assembly theories and aligns with observations from other cross-kingdom symbiotic systems. Conceptually, the “function-first, taxonomy-stochastic” framework indicates that functional diversity in the phycosphere saturates rapidly, while taxonomic diversity remains open-ended. Practically, recognizing that multiple taxa can fulfill the same functional roles enables more flexible and resilient design of synthetic algal-microbial consortia for ecological and industrial applications. Extending this work to other phytoplankton hosts and integrating multi-omics approaches will be essential to generalize the model, quantify the balance between stochastic and deterministic forces, and predict microbial community responses to environmental change.

## MATERIALS AND METHODS

### Isolation and incubation of *Skeletonema* strains

Samples were collected from Tongan Bay, Xiamen, China (N24°32′13″, E118°13′58″). Single putative *Skeletonema* chains were isolated by micropipette and washed with sterile seawater before culture to remove impurities and free-living microorganisms, thereby retaining only phycosphere-associated microorganisms (40). All *Skeletonema* strains were cultured in f/40-Si medium (41) prepared with natural seawater (salinity 29–30) that was pre-filtered through a 0.22 μm membrane filter (Millipore, Billerica, MA, USA) and autoclaved at 121°C for 20 min. Cultures were maintained in triplicate at 22.0°C ± 0.5°C under a 12-h light:12-h dark cycle with cool white fluorescent illumination at a photon flux density of 80 μmol/m^2^/s.

### Detection of inorganic nutrients

A 20 mL aliquot of the culture solution was collected on days 2, 4, 6, and 8 of the incubation and filtered through a pre-combusted (450°C, 4 h) GF/F glass fiber filter (0.7 μm pore size; Whatman, UK). Concentrations of NO_3_^−^-N, PO_4_^3−^-P, and SiO_3_^2−^-Si were then determined by spectrophotometry with AutoAnalyzer (Bran and Luebbe AA3, Germany).

### Genomic DNA extraction

Algal cells and their associated microorganisms were collected on days 2, 4, 6, and 8 of incubation by filtration of 50 mL of culture through a 0.22 μm polyethersulfone (PES) membrane filter (Millipore, Billerica, MA, USA) and stored at −80°C until DNA extraction. Additionally, 500 mL of seawater from the sampling site was filtered through a PES membrane during field sampling to obtain total environmental DNA. DNA was extracted following the protocol of Urakawa et al. (42) with slight modifications. Filters containing algal and microbial cells were placed in bead-beating tubes (Lysing Matrix E; MP Biomedicals) with 350 μL phenol-chloroform-isoamyl alcohol (25:24:1, TE-saturated, pH 8.0) and 350 μL TENS buffer (100 mmol/L Tris-HCl, pH 8.0; 40 mmol/L EDTA; 200 mmol/L NaCl; 2% SDS). Cells were disrupted in an MP FastPrep homogenizer at speed 6 for 40 s and centrifuged at 14,000 × *g* for 5 min. The aqueous phase was transferred to a 2 mL microcentrifuge tube, mixed with 300 μL 7.5 mol/L ammonium acetate by inversion, and extracted with an equal volume of chloroform. After thorough mixing, samples were centrifuged at 14,000 × *g* for 5 min. The supernatant was transferred to a 5 mL tube, mixed with 0.1 volumes of 3 mol/L sodium acetate and 2 volumes of 95% ethanol, and incubated on ice for 20–30 min. The mixture was then transferred to a spin column (Microcon YM-50; Millipore) and washed twice with ice-cold 70% ethanol. DNA was eluted with 50 μL DNase-free water, and concentrations were measured using a NanoDrop spectrophotometer (Thermo Scientific, USA).

### Identification of *Skeletonema* strains

The large subunit ribosomal DNA (LSU rDNA) was amplified using 2× Premix Taq (Takara Biotechnology, Dalian, China) with primers D1R: 5′-ACCCGCTGAATTTAAGCATA-3′ and D2C: 5′-CCTTGGTCCGTGTTTCAAGA-3′ (43). PCR products were sequenced using Sanger sequencing by Sangon Biotech Co., Ltd. (Xiamen, China). The resulting sequences were identified through BLASTn searches against the NCBI nucleotide database, and homologous sequences were retrieved for phylogenetic analysis. A neighbor-joining phylogenetic tree was constructed using MEGA X v10.0.5, and the resulting tree was visualized with FigTree v1.4.4.

### Amplicon sequencing and bioinformatic analysis of the phycosphere microbiome

The V3-V4 hypervariable region of the bacterial 16S rRNA gene was amplified from each DNA extract using primers 338F (5′-ACTCCTACGGGAGGCAGCAG-3′) and 806R (5′-GGACTACHVGGGTWTCTAAT-3′) (44). PCR amplification was confirmed by electrophoresis on a 1% agarose gel and purified using a Gel Extraction Kit (TIANGEN, China). Each sample was indexed with unique barcodes, and the purified amplicons were sequenced on an Illumina MiSeq platform (PE250) at Suzhou GENEWIZ Bio-pharm Technology Co., Ltd. (Suzhou, China). The raw sequencing data have been deposited in the Sequence Read Archive (SRA) under accession number PRJNA1205896. Raw reads were quality-filtered using Trimmomatic v0.39, and amplicon sequence variants (ASVs; sequences clustered at 100% similarity) were inferred using the DADA2 plugin in QIIME2 v2023.5 (45). Taxonomic annotation of all ASVs was performed by BLAST search against the SILVA database (Release 132), with assignments at the domain, phylum, class, order, family, genus, and species levels (46).

Given a set of 16S rDNA sequences from a sample, it is possible to identify the most closely related organisms with available sequenced genomes, enabling the inference that their associated functions are likely present in the sampled microbiome (47). PICRUSt2 is an algorithm and software package designed to predict functional profiles from 16S rDNA sequences by leveraging reference phylogenies to estimate the relative functional contributions of closely related sequenced genomes (48). In this study, the functional prediction of phycosphere microbial communities was explored using the PICRUSt2 algorithm via the QIIME2 plugin, with KEGG database annotations.

### Metagenomic sequencing and bioinformatic analysis of the phycosphere microbiome

For metagenome sequencing, total DNA extracts collected at day 4 from each *Skeletonema* were used. DNA quality and quantity were assessed prior to library construction. Metagenomic sequencing libraries were prepared following the manufacturer’s standard protocols and sequenced by Biomarker Technologies Corporation (Beijing, China). Shotgun metagenomic sequencing was performed on an Illumina NovaSeq 6000 platform, generating 150 bp paired-end reads. The raw sequencing data have been deposited in the Sequence Read Archive (SRA) under accession number PRJNA1205896. Raw paired-end reads were quality-filtered and trimmed using fastp v0.23.2 to remove adapters, low-quality bases, and short reads (<50 bp). High-quality reads were assembled *de novo* using MEGAHIT v1.2.9 with default parameters. Contigs were annotated with Prokka v1.14.6 in metagenome mode for gene prediction. Contigs longer than 1 Mb were truncated, and redundant sequences were clustered at 95% identity using CD-HIT v4.8.1 to generate non-redundant gene catalogs. Gene abundances were quantified by mapping clean reads to the non-redundant genes using Salmon v1.10.0. Taxonomic classification of predicted genes was performed with Kraken2 v2.1.2 against a custom database including bacteria, archaea, viruses, and eukaryotes. Functional annotation was conducted with eggNOG-mapper v2.1.9, assigning KEGG and GO terms for each predicted protein.

### Statistical analysis

Alpha diversity, including Richness, Evenness, and Phylogenetic Diversity (PD), was calculated using R v4.3.1 with the packages vegan and picante, and visualized with OriginPro v9.0. Differences in alpha diversity among phycosphere microbial communities were tested by one-way ANOVA using SPSS v22.0. Principal coordinate analysis (PCoA) based on Bray-Curtis and Jaccard distances was performed to visualize differences in both microbial community composition and functional profiles. Permutational multivariate analysis of variance (PERMANOVA) and analysis of similarities (ANOSIM) were conducted to assess the significance of group differences. These analyses were implemented in R using the packages vegan, dplyr, ggpubr, phyloseq, and ggplot2. Microbial species intersection analysis among different *Skeletonema* phycosphere communities was conducted using the UpSetR package in R to identify shared and unique taxa across samples. To identify microbial taxa significantly enriched in the phycospheres of different *Skeletonema* strains, linear discriminant analysis effect size (LEfSe) was performed with the Microeco package in R.

Co-occurrence network analysis was conducted using the Weighted Gene Correlation Network Analysis (WGCNA) package in R v4.3.1, based on Spearman correlation coefficients (|*r*| ≥ 0.75, *P* ≤ 0.01). Resulting networks were visualized in Gephi v0.10.0. To evaluate the stability of microbial networks, network robustness and vulnerability were calculated. The effect of species loss on network structure was assessed by simulating random node removal. The robustness in this study was regarded as when 50% of nodes were randomly removed, and results were based on 100 repetitions of the simulation. The vulnerability, which reflected the relative contribution of each node to the global efficiency, was represented by the maximal vulnerability of nodes in the network. Mantel tests were used to evaluate correlations between functional and taxonomic distance matrices. The potential contribution of stochastic processes to microbial community assembly was evaluated using a neutral community model, predicting the relationship between ASV detection frequency and their relative abundance across the metacommunity (49). This modeling was performed using the Hmisc, minpack.lm, and stats4 packages in R. The parameter *Nm*, representing the product of metacommunity size (N) and immigration rate (m), estimates dispersal between communities and governs the correlation between occurrence frequency and regional abundance. Model fit was assessed by the coefficient of determination (*R*^2^), and 95% confidence intervals for parameter estimates were calculated by bootstrapping with 1,000 bootstrap replicates.

## Data Availability

All raw sequencing data generated in this study have been deposited into the NCBI SRA under the accession number PRJNA1205896. All other data are available in the supplemental material.

## SUPPLEMENTAL MATERIAL

Supplemental material aem.00359-26-s0001.docx Figures S1 to S11; Tables S1 and S2.

ASM does not own the copyrights to Supplemental Material that may be linked to, or accessed through, an article. The authors have granted ASM a non-exclusive, world-wide license to publish the Supplemental Material files. Please contact the corresponding author directly for reuse.
