# Supplementary material for: Functional determinism amid taxonomic stochasticity: insights into rules governing the assembly of algal-microbial symbioses
Source: Appl Environ Microbiol. 2026 Mar 31;92(4):e00359-26. doi: 10.1128/aem.00359-26 (PMC13101525; doi:10.1128/aem.00359-26)
Supplement: Supplemental material — Figures S1 to S11; Tables S1 and S2. [file aem.00359-26-s0001.docx]

**Functional Determinism Amidst Taxonomic Stochasticity: Insights into rules governing the assembly of algal-microbial symbioses**

Tian Deng^1^, Huan Wang^1^, Shu-Feng Zhang^1,2^, Xin-Yao Wu^1^, Ze-Sheng Yang^1^, Da-Zhi Wang^1^, Yue Zheng^1, 3^ *

^1^ State Key Laboratory of Marine Environmental Science/College of the Environment and Ecology, Xiamen University, Xiamen 361102, China

^2^ Fujian Ocean Innovation Center, Xiamen 361102, China

^3^ Key Laboratory of the Ministry of Education for Coastal and Wetland Ecosystems, Xiamen University, Xiamen 361102, China

* The corresponding author: Yue Zheng, [yzheng@xmu.edu.cn](mailto:yzheng@xmu.edu.cn)


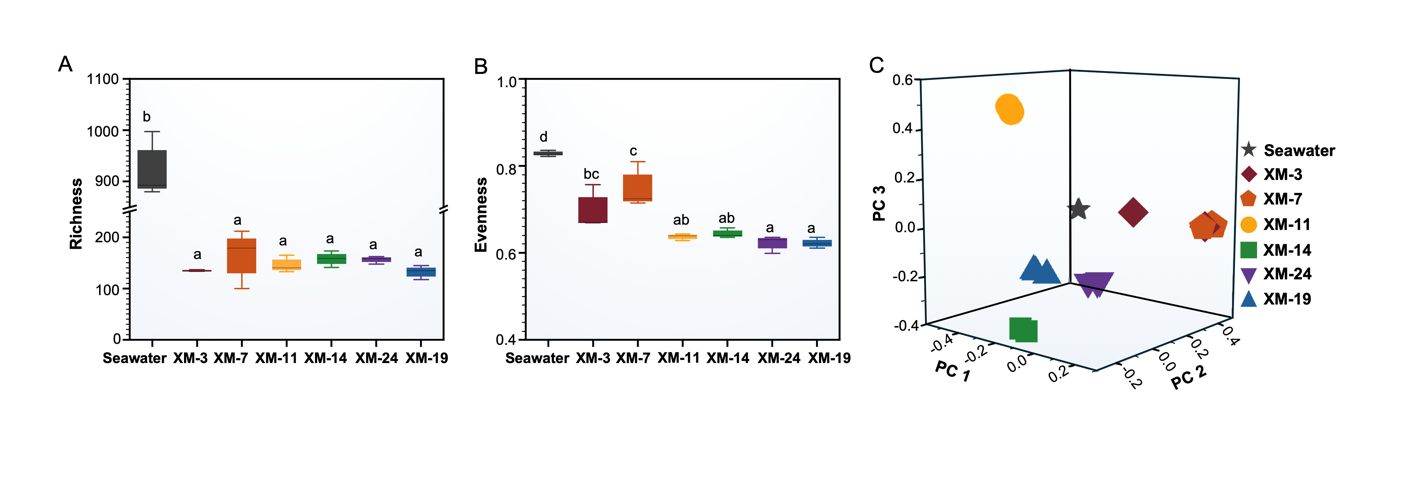


**Fig. S1** Diversity of phycosphere microbial communities of six *Skeletonema* strains and ambient seawater. **A** Richness. **B** Evenness **C** Beta diversity of phycosphere microbial communities in the six *Skeletonema* strains and ambient seawater, based on Jaccard PCoA.

**Fig. S2** Taxonomic composition of phycosphere microbiota associated with six *Skeletonema* strains and ambient seawater at different taxonomic levels.

**Fig. S3** Proportions (pie charts) and cumulative relative abundances (bar charts) of shared and unique ASVs in each sample. “Shared ASVs” refer to those present in all six *Skeletonema* strains; “Unique ASVs” denote those exclusively detected in a single strain; “Others ASVs” represent ASVs shared by 2-5 strains.

**Fig. S4** Evolutionary relationships of others ASVs (shared by several *Skeletonema* strains, top 30) and their average relative abundances in each *Skeletonema* strain (heatmap values represent average relative abundance).

**Fig. S5** Histogram of LDA (Linear Discriminant Analysis) scores for microbial genera differentially enriched among the six *Skeletonema* strains. The analysis was performed using an FDR-adjusted p value cutoff of 0.01 and a LDA score threshold of 3.

**Fig. S6** The alpha diversity of different growth stages of six *Skeletonema* strains.

**Fig. S7** Average relative abundances of predicted functional profiles of phycosphere microbial communities at KEGG level 2. (OS：Organismal Systems，HD：Human Disease，GIP：Genetic Information Processing，EIP：Environmental Information Processing，CP：Cellular Processes，M：Metabolism).

**Fig. S8** **A** Relative abundance of the top 20 KEGG pathways predicted from 16S rRNA gene sequences using PICRUSt2. The “top 20” pathways were selected based on the total relative abundance summed across all samples (all strains and all time points). The x-axis represents sampling time. **B** Relative abundances of the top 20 microbial genera, ranked by their total relative abundance across all samples. The x-axis represents sampling time.

**Fig. S9** Relative abundance of major microbial group (in phylum, class, order and family level) of phycosphere microbial communities of six *Skeletonema* strains.

**Fig. S10** **A** PCoA of taxonomic composition at the ASV level (Bray-Curtis distance). **B** PCoA of functional composition based on KEGG Orthology (Bray-Curtis distance)**. C** PCoA of taxonomic composition at the ASV level (Jaccard distance). **D** PCoA of functional composition based on KEGG Orthology (Jaccard distance).


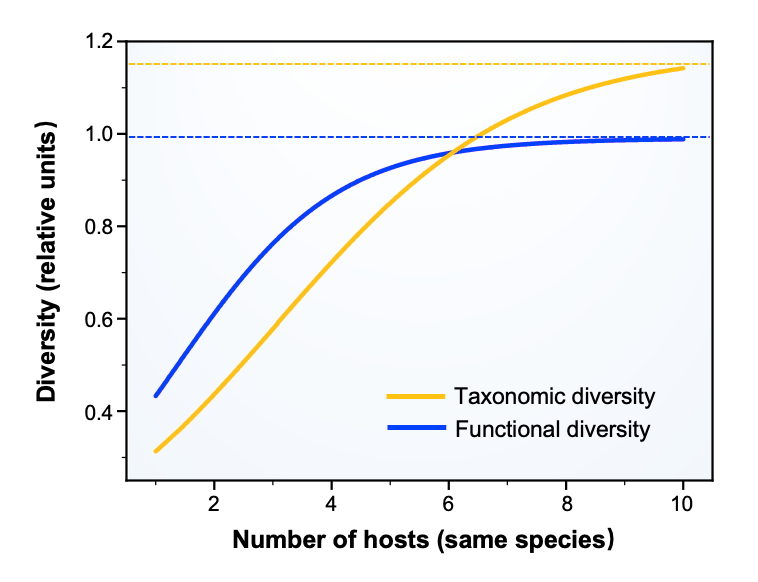


**Fig. S11** Conceptual model of functional and taxonomic diversity patterns in phycosphere microbiota.

**Table S1** the length and width of *Skeletonema*

|  | Length | Width |
| --- | --- | --- |
| XM-3 | 5.46 ± 0.31 | 3.91 ± 0.25 |
| XM-7 | 6.32 ± 0.66 | 5.43 ± 0.37 |
| XM-11 | 6.96 ± 0.99 | 5.16 ± 0.28 |
| XM-14 | 5.73 ± 0.53 | 4.18 ± 0.22 |
| XM-24 | 6.83 ± 0.57 | 5.20 ± 0.23 |
| XM-19 | 6.67 ± 1.12 | 4.48 ± 0.31 |

**Table S2** Statistical analysis of PCoA of different growth stages of *Skeletonema* strains

|  | ANOSIM | |  | PERMANOVA | | |
| --- | --- | --- | --- | --- | --- | --- |
|  | R | P |  | R^2^ | F | P |
| XM-3 | 0.136 | 0.229 |  | 0.417 | 1.90 | 0.1916 |
| XM-7 | 0.963 | 0.001 |  | 0.920 | 30.60 | 0.0002 |
| XM-11 | 0.904 | 0.001 |  | 0.873 | 18.29 | 0.0003 |
| XM-14 | 0.66 | 0.002 |  | 0.751 | 8.03 | 0.0012 |
| XM-24 | 0.071 | 0.262 |  | 0.354 | 1.46 | 0.2643 |
| XM-19 | 0.821 | 0.001 |  | 0.948 | 48.95 | 0.0004 |
